# LncRNA LINC00461 exacerbates myocardial ischemia–reperfusion injury via microRNA-185-3p/Myd88

**DOI:** 10.1186/s10020-022-00452-1

**Published:** 2022-03-10

**Authors:** Feng Gao, Xiaochen Wang, Tingting Fan, Zhidan Luo, Mengqing Ma, Guangquan Hu, Yue Li, Yi Liang, Xianhe Lin, Banglong Xu

**Affiliations:** 1 Department of Cardiology, Economic Development District, Second Affiliated Hospital of Anhui Medical University, No.678 Furong Road, Hefei, 230601 Anhui China; 2 Department of Geriatrics, Chongqing People’s Hospital, Chongqing, 400013 China; 3 Department of Cardiology, The First Affiliated Hospital of Anhui Medical University, No.218 Jixi Road, Shushan District, Hefei, 230022 Anhui China; 4 Center for Cardiovascular Regeneration, Houston Methodist Research Institute, 6670 Bertner Ave, Houston, TX 77030 USA

**Keywords:** Long non-coding RNA LINC00461, microRNA-185-3p, Myeloid differentiation primary response gene 88, Myocardial ischemia–reperfusion, Apoptosis, Oxidative stress, Cardiac function

## Abstract

**Objective:**

Long non-coding RNAs (lncRNAs) play critically in the pathogenesis of myocardial ischemia–reperfusion (I/R) injury. Thus, it was proposed to investigate the mechanism of LINC00461 in the disease through mediating microRNA-185-3p (miR-185-3p)/myeloid differentiation primary response gene 88 (Myd88) axis.

**Methods:**

miR-185-3p, LINC00461 and Myd88 expression in mice with I/R injury was measured. Mice with I/R injury were injected with the gene expression-modified vectors, after which cardiac function, hemodynamics, myocardial enzyme, oxidative stress, and cardiomyocyte apoptosis were analyzed.

**Results:**

I/R mice showed LINC00461 and Myd88 up-regulation and miR-185-3p down-regulation. Down-regulating LINC00461 or up-regulating miR-185-3p recovered cardiac function, reduced myocardial enzyme levels, and attenuated oxidative stress and cardiomyocyte apoptosis in mice with I/R. miR-185-3p overexpression rescued the promoting effect of LINC00461 upregulation on myocardial injury in I/R mice.

**Conclusion:**

LINC00461 knockdown attenuates myocardial I/R injury via elevating miR-185-3p expression to suppress Myd88 expression.

**Supplementary Information:**

The online version contains supplementary material available at 10.1186/s10020-022-00452-1.

## Introduction

Myocardial ischemia–reperfusion (I/R) injury is a main factor leading to coronary artery disease-related morbidity and mortality (Li et al. 2019a). Thrombolysis, cardiac surgery and primary angioplasty are effective treatment methods widely utilized in clinical practice to restore blood flow in the ischemic myocardium (Yao et al. 2019). However, sudden recovery of blood flow may lead to additional cardiovascular trauma, named reperfusion injury (Huang et al. 2019). Myocardial I/R injury transforms conduction system and excitability of cardiac muscles, causes systolic function and cardiac diastolic damage, and triggers severe myocardial dysfunction and injury (Zhao et al. 2018). Although with great efforts, the molecular mechanisms implicated in the initiation and development of myocardial I/R injury were not fully understood (Zhang et al. 2017c), which ask for extensive exploration for the control of myocardial I/R injury.

Long non-coding RNAs (lncRNAs) become new modulators in various biological processes, containing RNA splicing and epigenetic regulation (Wang et al. 2016). Commonly, ncRNA annotation, expression profile, structural and molecular changes, and interaction with other molecules are involved in cardiovascular diseases (Balamurali and Stoll 2020). A recent study has elucidated that lncRNA LINC00461 is associated with the survival of renal cell carcinoma patients (Chen et al. 2019). Another study has reported that LINC00461 facilitates the development of breast cancer and glioma (Ji et al. 2019). miRs are single-stranded small non-coding RNAs that participate in many biological processes (Liu et al. 2019). It has been accepted that myocardial miRs are essential for the phenotypic transformation of myofibroblasts after infarction (Morelli et al. 2019; Wang et al. 2020), and miR signature may of utility for predicting heart failure (Charrier et al. 2019). Especially, endogenous inhibition of miR-185 could facilitate the recovery of cardiac function in mice after myocardial infarction (Li et al. 2019), and circulating miR-185 may be a prognostic biomarker of patients with dilated cardiomyopathy (Yu et al. 2016). Myeloid differentiation primary response gene 88 (Myd88) is a key regulator in the innate immune system (Lefort et al. 2019). It has been presented that bone marrow Myd88 mediates ischemic myocardial injury and neutrophil function (Feng et al. 2010), and Myd88 knockdown could protect against myocardial injury based on a rat model of I/R (Zhang et al. 2017b). In this work, we discussed the effect of LINC00461/miR-185-3p/Myd88 axis on myocardial I/R injury.

## Materials and methods

### Ethics statement

Animal experimental protocol was complied with the Guide for the Care and Use of Laboratory Animal by International Committees, and was approved by the Institutional Animal Care Use Committee of The First Affiliated Hospital of Anhui Medical University.

### Experimental animals

Male healthy C57BL/6 mice (10–12 weeks old, 20–30 g) were bought from the experimental animal center of Anhui Medical University (Anhui, China). The mice were fed for 1 w in a clean animal house at 22–25 °C, during which water and food were supplied with normal circadian rhythm. Twenty mice were divided into the sham group and I/R group, and the rest 70 mice were treated according to the successful modeling method.

### Modeling of I/R mice

Fasted for 8 h, mice were anesthetized with 1% pentobarbital sodium at 60 mg/kg (Sigma, Santa Clara, CA, USA) and fixed in a supine position with the skin of the regiones colli anterior cut off after disinfection. The tissues and muscles were separated and trachea was exposed; trachea cannula was inserted from the mouth and connected with a respirator. The mice were placed in the right lateral position, and a 2-cm longitudinal incision was made on the left side of the epidermis of the fifth costal space. The ectopectoralis and entopectoralis were separated, and the fourth costal space was exposed. The fourth costal space was impaled by mosquito forceps, the mediastinum to the left was penetrated, and then the heart was extruded. At 0.5 cm from the lower edge of the left atrial appendage, the left coronary artery was ligated with a 6-0 suture. Left ventricular anterior wall whitening or ST segment elevation in the electrocardiogram (ECG) indicated the successful ligation. After ischemia of 30 min, the ligation was loosened. The ST-segment raised in the ECG (ST segment recovery or a change significantly different from that of the previous waveform) within 5 min was noted as a reperfusion success. The heart was then re-located to the chest and the contralateral thoracic cavity was pressed with hand to avoid pneumothorax. After the operation, the mice were put back into the animal cage and the vital signs were observed. Thoracotomy was performed in the sham group, but the left coronary artery was not ligated. 

### Grouping and treatment

Seventy I/R mice (10 mice/group) were respectively injected with normal saline, LINC00461 siRNA (si-LINC00461), siRNA -negative control (si-NC), miR-185-3p mimic, mimic NC, miR-185-3p mimic + overexpression (OE)-LINC00461 vector, or mimic NC + OE-LINC00461 through the tail vein 24 h before modeling. All the vectors were bought from GenePharma (Shanghai, China).

### Echocardiography

Acuson Sequoia 512 Ultrasound System (Siemens Medical Solutions USA, Inc., Mountain View, CA, USA) was utilized for assessing cardiac function. Left ventricular ejection fraction (LVEF) and left ventricular fractional shortening (LVFS) were calculated.

### Hemodynamic detection

After intraperitoneal injection of 1% pentobarbital sodium in mice, the right common carotid artery was separated. A PE50 artery catheter was inserted into the left ventricle through the right common carotid artery. The left ventricular end diastolic pressure (LVEDP), together with maximum left ventricular pressure rising and dropping rate (± dp/dt max) were measured by MP150 biological function experiment system (BIOPAC Systems, Inc, CA, USA).

### Reverse transcription quantitative polymerase chain reaction (RT-qPCR)

Trizol (Invitrogen, CA, USA) was utilized for extracting total RNA in myocardial tissues. The concentration and quality of RNA were determined by NanoDrop2000 (Thermo Fisher Scientific, MA, USA). Expression of LncRNA and mRNA was quantified using PrimeScript™ RT reagent Kit (TaKaRa, Dalian, China)  and TB Green™Premix Ex Taq™ II (TaKaRa). miRNA expression was calculated using miRcute Plus miRNA First-Strand cDNA Kit and miRcute Plus miRNA qPCR Kit (SYBR Green) (Tiangen) (Fu et al. 2020). The primers were composed by BGI Co. (Shenzhen, Guangdong, China) (Additional file 3: Table S1). U6 was the endogenous control of miR-185-3p while glyceraldehyde phosphate dehydrogenase (GAPDH) was that of LINC00461 and Myd88. Data were reckoned by 2^−ΔΔCt^ method (Livak and Schmittgen 2001).

### Western blot analysis

The total protein in the myocardial tissue of mice was extracted and quantified by bicinchoninic acid method (Boster, Hubei, China). After sodium dodecyl sulphate polyacrylamide gel electrophoresis, the protein was transferred to a polyvinylidene fluoride membrane and sealed with 5% bovine serum albumin. The membrane was cultured with primary antibodies Myd88 (1:1000), Bcl-2 (1:2000), Bax (1:2000) and GAPDH (1:3000) (all from Abcam, MA, USA) and with corresponding secondary antibody (MTBio, Shanghai, China). The plots were developed by chemiluminescence reagent, observed by Bio-rad Gel Doc EZ imager (Bio-rad, CA, USA) and analyzed by ImageJ software (Gao et al. 2015).

### RNA pull-down assay

Three different biotin-labeled miRNA sequences were designed: wild type (WT) miR-185-3p (Bio-miR-185-3p-WT), mutant type (MUT) miR-185-3p (Bio-miR-185-3p-MUT, the sequence complementary to LINC00461 was mutated), and a random miRNA (Bio-NC). The miRNA sequences were transfected into cells when the confluence was 80–90% was transfected, respectively. After 48 h, the cell lysate was hatched with M-280 streptavidin-coated magnetic beads (Sigma), and the protein-nucleic acid complex was eluted and lysed using Trizol to extract RNA, thus to detect LINC00461 expression by RT-qPCR.

### Dual luciferase reporter gene assay

The putative miR-185-3p binding sites were assessed in LINC00461/Myd88 3′-UTR. The pMIR-REPORT™ (RiboBio), covering wild type (WT) or mutant (MUT) LINC00461/Myd88 3′-UTR sequences was employed for carrying out the dual-luciferase reporter gene assay.  WT or MUT LINC00461/Myd88 3′-UTR vector,  along with miR-185-3p mimic or its NC was transfected into 293T cells at 70% confluence via Lipofectamine 2000 (Invitrogen). The luciferase activity was detected by Dual-Luciferase^@^ Reporter Assay System kit (Promega Corporation, WI, USA) 48 h later.

### Hematoxylin–eosin (HE) staining

The myocardial tissue of mice was prepared into 5 μm paraffin slices, baked in an oven at 60℃ for 20 min, permeabilized and hydrated with 95%, 80% and 75% ethanol for 2 min in turn. Then, the tissue was cleaned with running water, dyed with hematoxylin and treated with ammonia. After dyeing with eosin for 1 min, the tissue was blocked with neutral gum and examined under a light microscope.

### Picric acid-Sirius red staining

Myocardial tissue sections were dewaxed by turpentine I, II solution for 15 min, respectively, then hydrated by 100% ethanol I, II, 95%, 90%, 80% and 75% ethanol and distilled water for 5 min, respectively, placed in hematoxylin solution for 2–3 min, rinsed with clear water for 30 min, and dehydrated with 75%, 80%, 95% and 100% for 3 min, respectively. The sections that were cleared with xylene and sealed with neutral gum were subjected to analysis under a light microscope.

### Terminal deoxynucleotidyl transferase-mediated dUTP-biotin nick end-labeling (TUNEL) staining

The myocardial tissue sections in each group were taken and operated as per the instructions of the TUNEL Apoptosis Detection Kit (Roche Diagnostics, Indianapolis). Five fields of view were selected in a random manner under each slice microscope for viewing the apoptotic cells in the field of view and count. The ratio of apoptotic cells in the statistical field was a ratio of the total number of cardiomyocytes.

### Serum index detection

Serum was collected after centrifugation at 3000 r/min for 10 min. Using lactate dehydrogenase (LDH) kit, creatine kinase-MB (CK-MB) kit, cardiac troponinI (cTn-I) kit and nitric oxide (NO) kit (all from NanJing JianCheng Bioengineering Institute, Nanjing, China), the serum sample was reacted with corresponding reagent. With the optical density (OD) determined by a microplate reader, LDH, CK-MB, cTn-I and NO contents were calculated.

### Detection of myocardial tissue index in I/R mice

Myocardial tissue samples were homogenized in the pre-cooled 0.9 NaCl (1 mL/100 mg) at 1:9. The homogenate was placed in pre-cooled homogenate medium (0.01 M Tris–HCl, 0.0001 M ethylene diamine tetraacetic acid-2Na, 0.01 M sucrose, 0.8% NaCl, pH 7.4) to prepare 10% homogenate. Reactive oxygen species (ROS) and malondialdehyde (MDA) content, as well as superoxide dismutase (SOD) activity were determined by the kits (JianCheng Bioengineering Institute). For MDA, the wavelength of 532 nm was selected, and for SOD activity, the wavelength of 550 nm was determined.

### Statistical analysis

All data were processed by SPSS 21.0 software (IBM Corp. Armonk, NY, USA) and GraphPad Prism 6.0 (GraphPad Software, San Diego, CA). Measurement data were interpreted as mean ± standard deviation. Comparisons between two groups were conducted by *t*-test. Comparisons among multiple groups were assessed by one-way analysis of variance (ANOVA) and Tukey’s post hoc test. *P* < 0.05 meant statistically significance.

## Results

### Up-regulating miR-185-3p or down-regulating LINC00461 improves cardiac function of I/R mice

It was examined that LVFS, LVEF, ± dp/dt max decreased and LVEDP raised in mice after I/R operation, indicating that the myocardial I/R model was successfully constructed. Up-regulating miR-185-3p or down-regulating LINC00461 increased LVFS, LVEF, ± dp/dt max and suppressed LVEDP. miR-185-3p overexpression reversed the effects of LINC00461 upregulation on LVFS, LVEF, ±dp/dt max and LVEDP in I/R mice (Additional file 1: Fig. S1A–E). Briefly, inhibiting LINC00461 or up-regulating miR-185-3p can improve cardiac function of mice after myocardial I/R injury.

### LINC00461 binds to miR-185-3p; LINC00461 is up-regulated while miR-185-3p is down-regulated in I/R mice

**Fig. 1:**
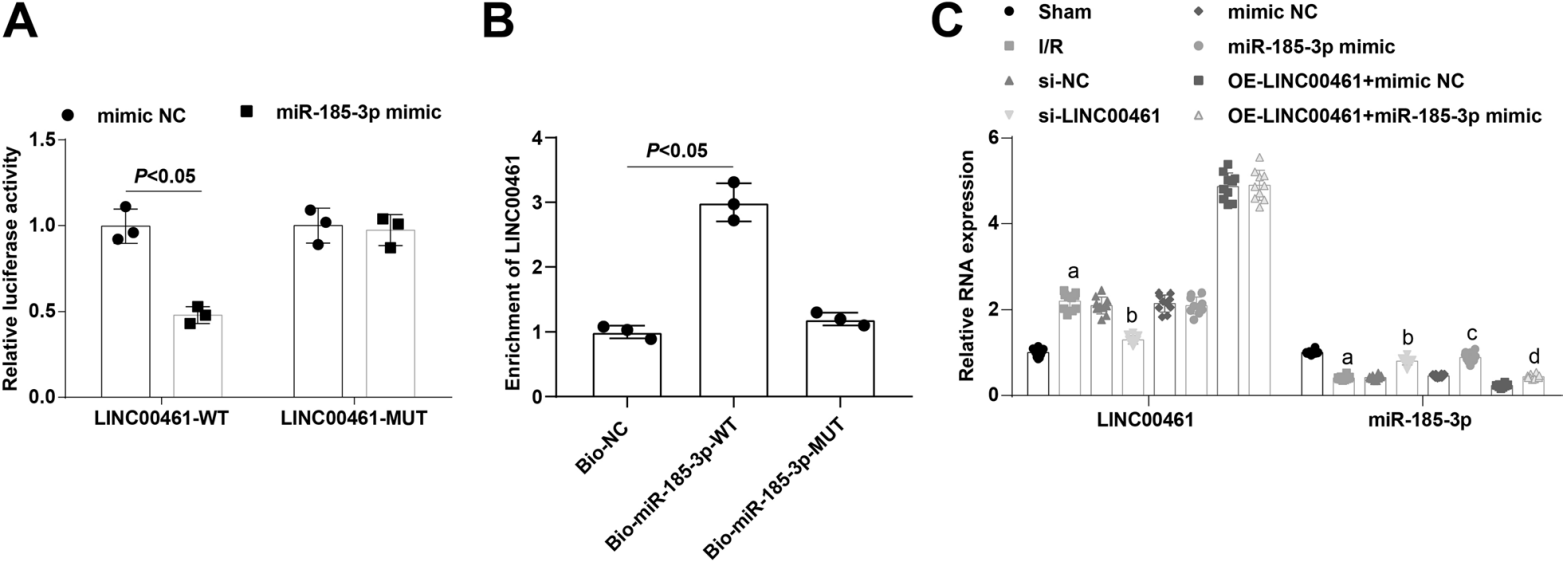
LINC00461 binds to miR-185-3p; LINC00461 is up-regulated while miR-185-3p is down-regulated in I/R mice. **A** Dual luciferase reporter gene assay verified the regulatory relationship between LINC00461 and miR-185-3p. **B** The relationship between LINC00461 and miR-185-3p in myocardial tissue by RNA-pull down assay. **C** Expression of LINC00461 and miR-185-3p in myocardial tissue of mice in each group. a *P* < 0.05 vs. the sham group. b *P* < 0.05 vs. the si-NC group. c *P* < 0.05 vs. the mimic-NC group. d *P* < 0.05 vs. the OE-LINC00461 + mimic NC group

The specific binding region of LINC00461 and miR-185-3p was predicted by the bioinformatics website RNA22 version 2.0 (http://cm.jefferson.edu/) (Additional file 2: Fig. S2A). Dual luciferase reporter gene assay showed that miR-185-3p mimic impaired the luciferase activity of WT LINC00461, while imposed no effect on that of MUT LINC00461 (Fig. 1A).

RNA pull-down assay revealed that LINC00461 expression raised by Bio-miR-185-3p-WT, and Bio-miR-185-3p-MUT had no distinct influence on LINC00461 expression (Fig. 1B), proving that miR-185-3p could pull down LINC00461.

RT-qPCR was utilized to detect LINC00461 and miR-185-3p expression in the myocardial tissue of mice, demonstrating that LINC00461 expression was enhanced and miR-185-3p expression was restrained in mice after I/R. LINC00461 down-regulation decreased LINC00461 expression while elevated miR-185-3p expression, and miR-185-3p restoration elevated miR-185-3p expression. LINC00461 overexpression-mediated expression of miR-185-3p was restored by miR-185-3p up-regulation (Fig. 1C). In brief, LINC00461 can combine with miR-185-3p to regulate miR-185-3p expression.

### MiR-185-3p targets Myd88 and Myd88 expression increases in I/R mice

**Fig. 2:**
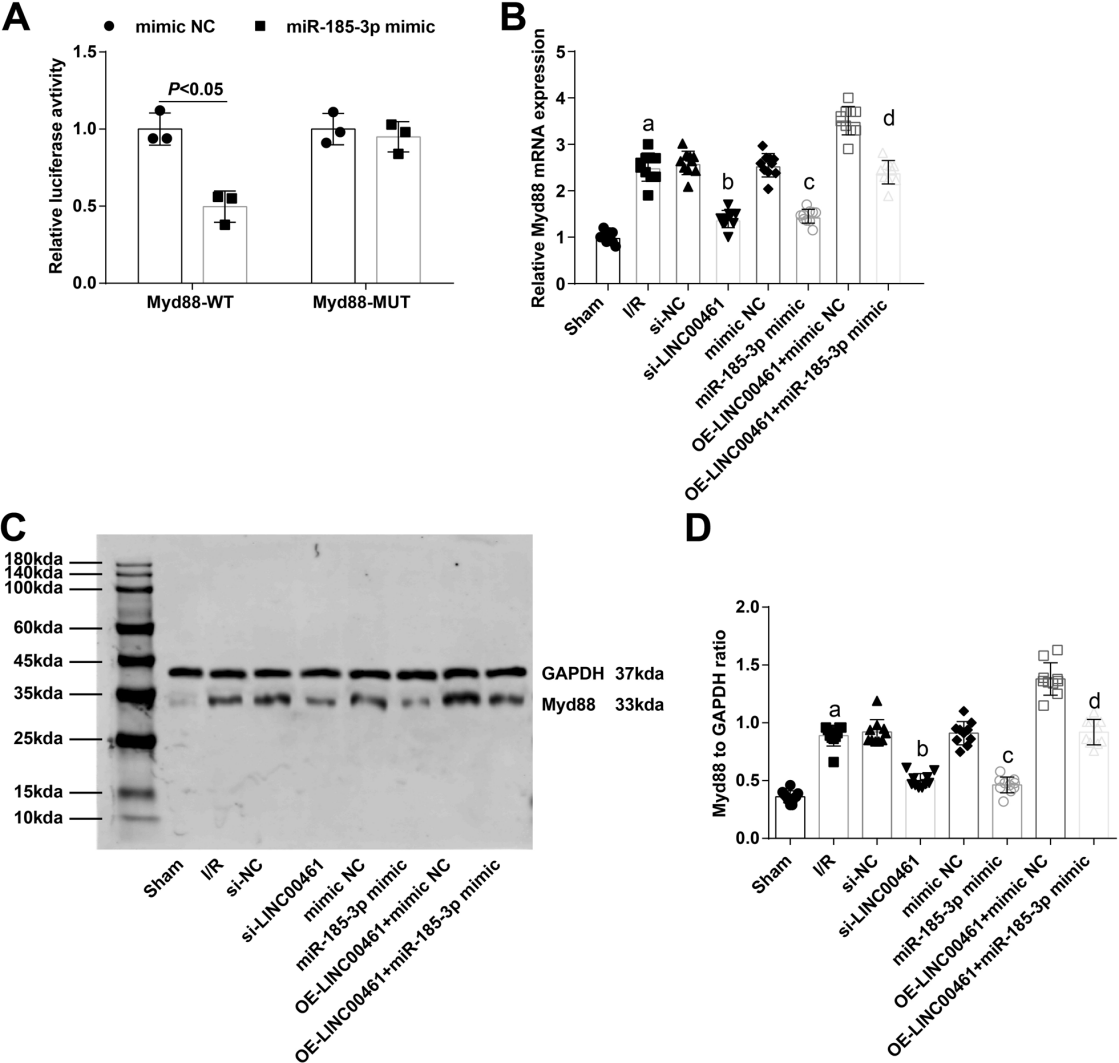
MiR-185-3p targets Myd88 and Myd88 expression increases in I/R mice. **A** The targeting relationship between Myd88 and miR-185-3p was verified by dual luciferase reporter gene assay. **B** Comparison of Myd88 mRNA expression in myocardial tissue of mice. **C** Protein bands of Myd88 of mice in each group. **D** Comparison of Myd88 protein expression in the myocardial tissue of each group. a *P* < 0.05 vs. the sham group. b *P* < 0.05 vs. the si-NC group. c *P* < 0.05 vs. the mimic-NC group. d *P* < 0.05 vs. the OE-LINC00461 + mimic NC group

The binding site of Myd88 and miR-185-3p was predicted by the bioinformatics website RNA22 version 2.0 (http://cm.jefferson.edu/) (Additional file 2: Fig. S2B). Myd88 was a target of miR-185-3p, which was verified by the outcome of luciferase reporter assay: the luciferase activity of Wt-Myd88 was reduced in miR-185-3p-overexpressed cells (Fig. 2A).

Western blot analysis and RT-qPCR detected Myd88 expression in the myocardial tissues, presenting that Myd88 mRNA and protein expression was elevated in mice after I/R, which could be suppressed when miR-185-3p was restored or LINC00461 was silenced. Moreover, it was exhibited that the levels of Myd88 promoted by overexpressed LINC00461 were reduced after miR-185-3p up-regulation (Fig. 2B–D).

### Up-regulating miR-183-3p or down-regulating LINC00461 alleviates the pathological injury of I/R mice

**Fig. 3:**
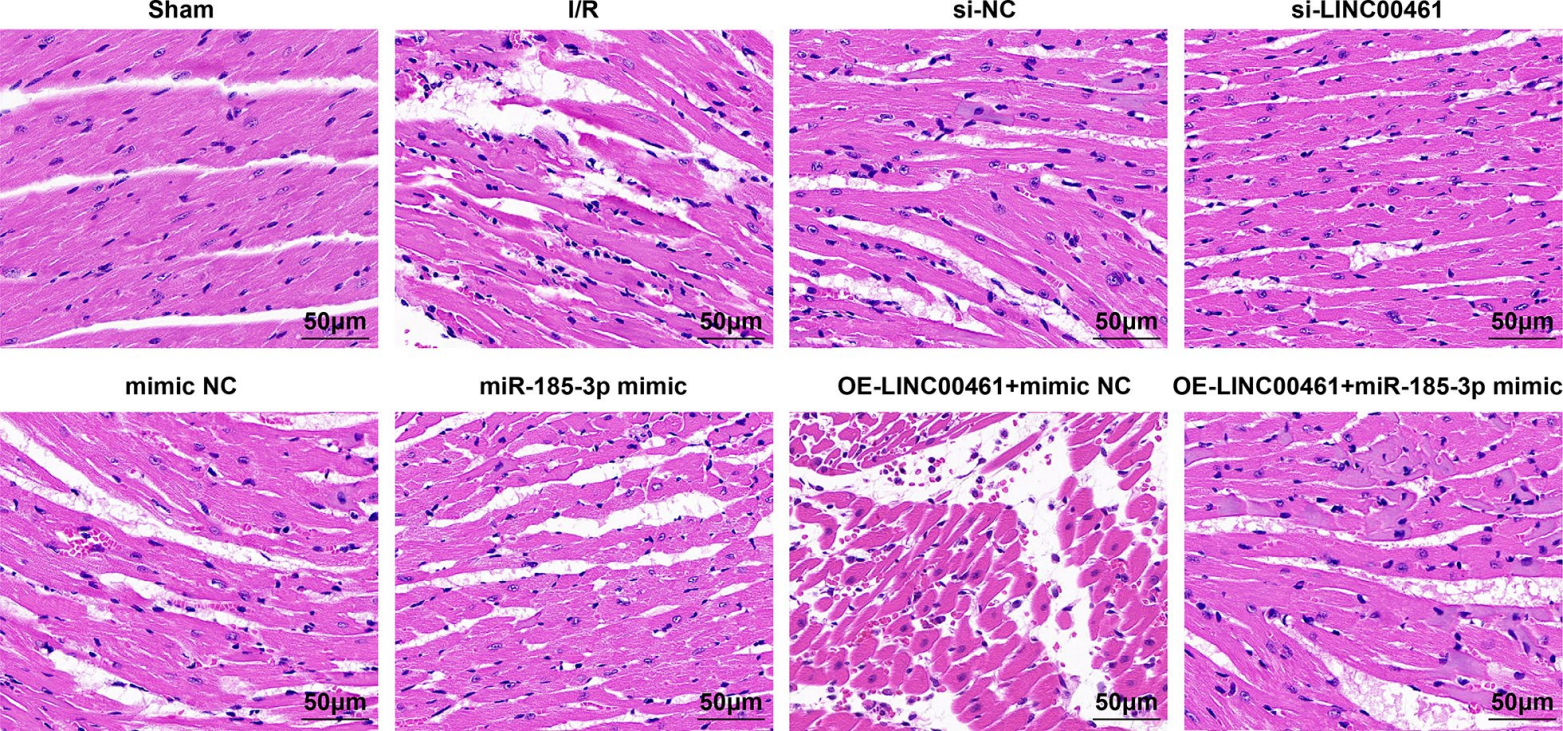
Up-regulating miR-183-3p and down-regulating LINC00461 alleviate the pathological injury of I/R mice. The changes of myocardial tissue structure in each group of mice by HE staining

HE staining revealed that in normal mice, myocardial fiber cells were arranged neatly and tightly, the cytoplasm and nucleus staining was uniform, with only a small amount of bleeding, mild edema and without necrosis, apoptosis, or inflammatory cell infiltration. In I/R mice, and I/R mice injected with si-NC, mimic NC or OE-LINC00461 + miR-185-3p mimic, broken myocardial fibers were loosely arranged with obvious intervals, serious bleeding and edema, obvious cell necrosis and serious infiltration of inflammatory cells. I/R mice injected with si-LINC00461 or miR-185-3p mimic showed neatly and tightly arranged myocardial fiber cells with less bleeding, mild edema, a small number of cell necrosis and inflammatory cell infiltration, and the overall condition was better than I/R mice. In I/R mice injected with OE-LINC00461 and mimic NC, aggravated myocardial fiber rupture, loosely arranged cells with obvious intervals, aggravated bleeding and edema, and severe cell necrosis and inflammatory cell infiltration were observed; the overall condition was severer than I/R mice (Fig. 3). Shortly, inhibiting LINC00461 or up-regulating miR-185-3p can improve tissue pathology after myocardial I/R injury in mice.

### Restoration of miR-185-3p or depletion of LINC00461 reduces myocardial fibrosis in I/R mice

**Fig. 4:**
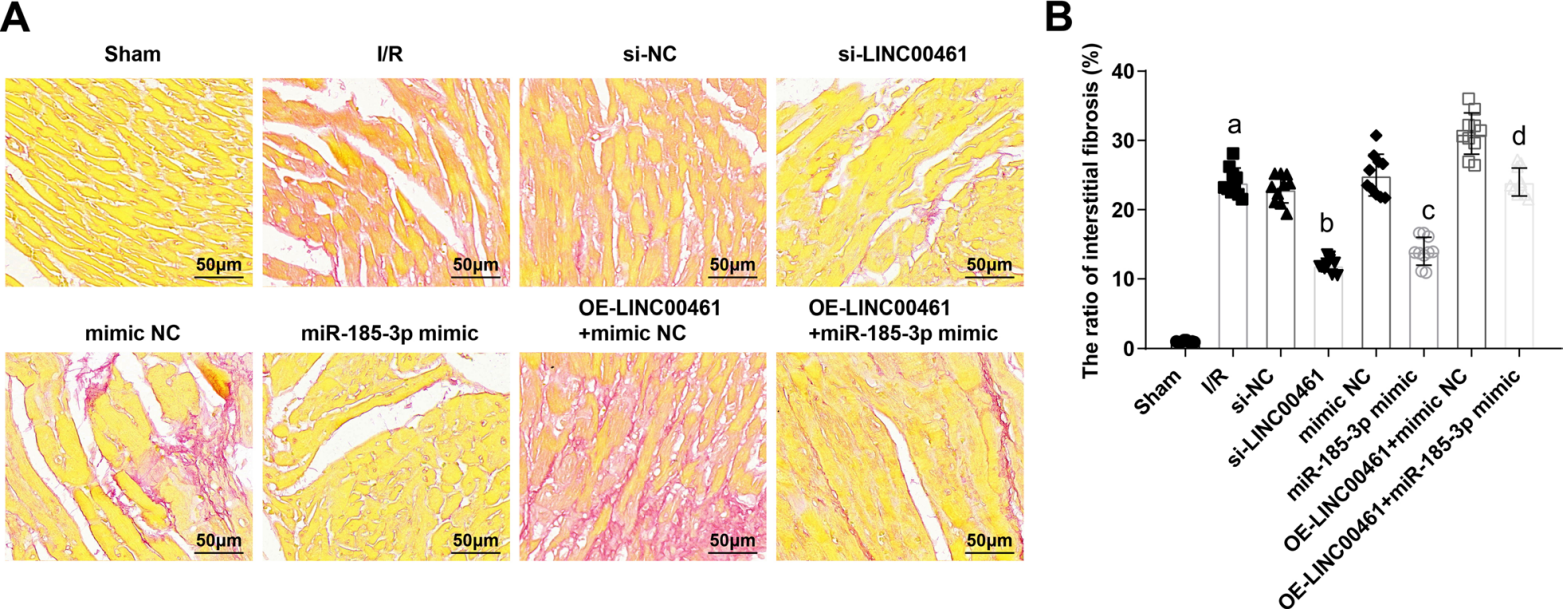
Restoration of miR-185-3p or depletion of LINC00461 reduces myocardial collagen hyperplasia and fibrosis in I/R mice. **A** Degree of myocardial fibrosis in each group of mice. **B** Comparison of myocardial fibrosis area in each group of mice. a *P* < 0.05 vs. the sham group. b *P* < 0.05 vs. the si-NC group. c *P* < 0.05 vs. the mimic-NC group. d *P* < 0.05 vs. the OE-LINC00461 + mimic NC group

Picric acid-Sirius red staining demonstrated that in I/R mice, the myocardial collagen was obviously proliferated and thickened, the cardiomyocytes were divided into a mesh-like shape, and fibrotic area was increased. Silencing LINC00461 or restoring miR-185-3p alleviated myocardial collagen hyperplasia and reduced the fibrotic area in I/R mice. LINC00461 overexpression-induced myocardial collagen hyperplasia and fibrotic area were alleviated by up-regulating miR-185-3p in I/R mice (Fig. 4A, B). Evidently, inhibiting LINC00461 or up-regulating miR-185-3p reduces fibrosis after myocardial I/R injury in mice.

### Restored miR-185-3p or depleted LINC00461 suppresses apoptosis of cardiomyocytes in I/R mice

**Fig. 5:**
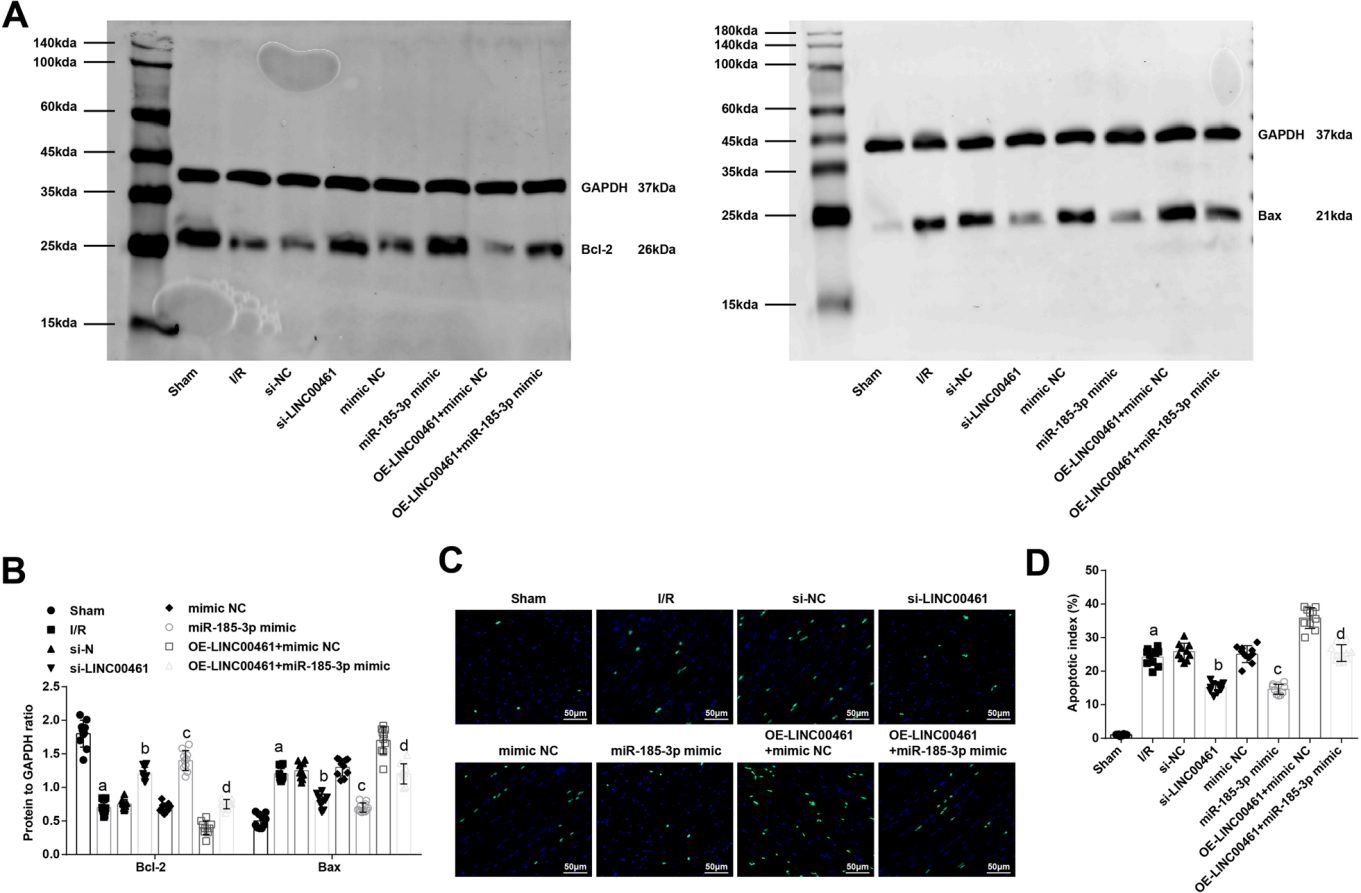
Restored miR-185-3p or depleted LINC00461 suppresses apoptosis of cardiomyocytes in I/R mice. **A**, **B** Comparison of Bcl-2 and Bax protein expression in the myocardial tissue of mice. **C**, **D** Comparison of cardiomyocyte apoptosis in the myocardial tissue of mice. a *P* < 0.05 vs. the sham group. b *P* < 0.05 vs. the si-NC group. c *P* < 0.05 vs. the mimic-NC group. d *P* < 0.05 vs. the OE-LINC00461 + mimic NC group

Western blot analysis tested Bcl-2 and Bax protein expression while TUNEL staining tested cell apoptosis. The results displayed that Bcl-2 expression reduced while Bax expression and cell apoptosis rate increased in I/R mice. The protein expression of the two indicators and cell apoptosis were converted in I/R-injured mice by LINC00461 suppression or miR-185-3p induction. Moreover, miR-185-3p restoration reduced the promoting impacts of LINC00461 overexpression on apoptosis in the myocardium of mice with I/R (Fig. 5A–D). Plainly, silencing LINC00461 or restoring miR-185-3p suppresses apoptosis in the myocardial tissue after I/R injury in mice.

### Up-regulating miR-185-3p or down-regulating LINC00461 suppresses LDH, CK-MB and cTnӀ as well as raises NO in I/R mice

**Fig. 6:**
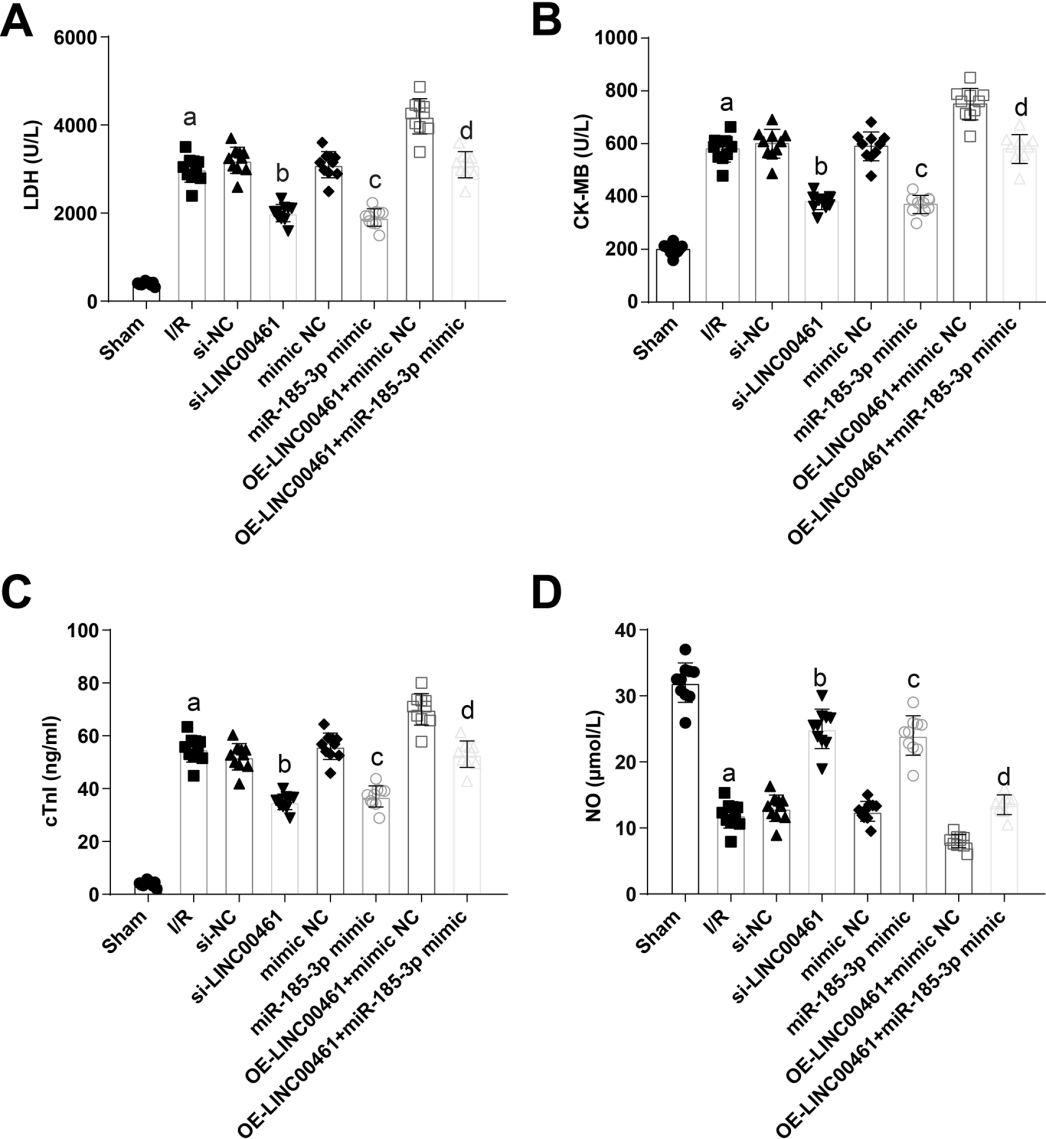
Up-regulating miR-185-3p or down-regulating LINC00461 decreases LDH, CK-MB and cTnӀ contents as well as increases NO content in I/R mice. **A** Content of LDH in serum of abdominal aorta of mice in each group. **B** Content of CK-MB in serum of abdominal aorta of mice in each group. **C** Content of cTnӀ in serum of abdominal aorta of mice in each group. **D** Content of NO in serum of abdominal aorta of mice in each group. a *P* < 0.05 vs. the sham group. b *P* < 0.05 vs. the si-NC group. c *P* < 0.05 vs. the mimic-NC group. d *P* < 0.05 vs. the OE-LINC00461 + mimic NC group

By analysis of serum sample collected from the abdominal aorta of mice, it was suggested that LDH, CK-MB and cTnӀ contents elevated and NO content decreased in I/R mice. The contents of the indicators were altered after silencing LINC00461 or restoring miR-185-3p. LINC00461 overexpression-mediated contents of the indicators could be restored by inducing the expression of miR-185-3p (Fig. 6A–D). To shortly conclude, decreased LINC00461 or increased miR-185-3p can improve myocardial enzyme after I/R injury in mice.

### Restoring miR-185-3p or silencing LINC00461 attenuates oxidative stress in I/R mice

**Fig. 7:**
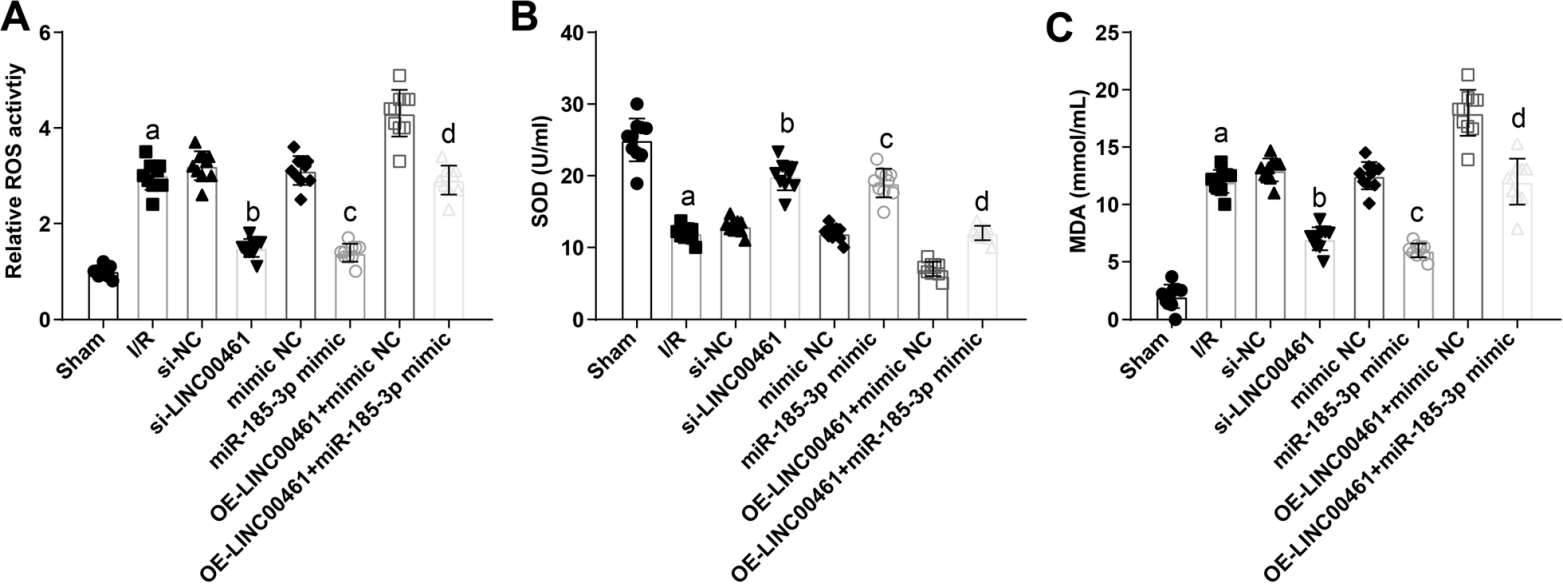
Restoring miR-185-3p or silencing LINC00461 decrease oxidative stress in the myocardial tissue of I/R mice. **A** Comparison of ROS levels in myocardial tissue of mice. **B** Comparison of SOD levels in myocardial tissue of mice. C, Comparison of MDA levels in myocardial tissue of mice. a *P* < 0.05 vs. the sham group. b *P* < 0.05 vs. the si-NC group. c *P* < 0.05 vs. the mimic-NC group. d *P* < 0.05 vs. the OE-LINC00461 + mimic NC group

Through analysis oxidative stress-related indicators in the myocardial tissue of mice, it was presented that ROS and MDA contents elevated and SOD activity impaired in I/R mice. Restoring miR-185-3p or silencing LINC00461 showed a great ability to constrain ROS and MDA contents and enhanced SOD activity. The promoting effect of LINC00461 overexpression on oxidative stress was suppressed after miR-185-3p was up-regulated (Fig. 7A–C). All in all, LINC00461 deficiency or miR-185-3p elevation can attenuate oxidative stress in the myocardial tissue after I/R injury in mice.

## Discussion

Myocardial I/R injury is a complicated pathophysiological process (Zheng et al. 2017). A previous study has discussed that lowly expressed lncRNA KCNQ1OT1 protects against myocardial I/R injury (Li et al. 2017). Another study has reported that miR-497 accelerates proliferation and suppresses cardiomyocyte apoptosis in myocardial I/R injury (Qin et al. 2018). Furthermore, it is revealed a critical role for MyD88-dependent signaling pathway during myocardial I/R injury while the regulation of the IL-1R/MyD88 interaction may be a strategy for managing myocardial ischemic injury (Cao et al. 2009). Our study was to decode LINC00461/miR-185-3p/Myd88 axis-related mechanism in myocardial I/R injury.

Our study provided evidence that LINC00461 and Myd88 expression increased while miR-185-3p expression decreased in I/R mice. Recently, a study has implicated that LINC00461 expression is elevated in hepatocellular carcinoma (HCC) tissues which is positively related to advanced stage and metastasis (Ji et al. 2019). Another study has presented that the increase of LINC00461 expression in breast cancer is related to tumor differentiation and TNM stage (Dong et al. 2019). It is reported that miR-185 expression is notably suppressed in cardiomyocytes in the process of cardiac hypertrophy caused by transverse aortic constriction (Kim et al. 2015). Similarly, a previous study has pointed out that miR-185-5p expression is markedly reduced in the heart of mice with myocardial infarction (Li et al. 2019). Also, it is examined that in acute ST-segment elevation myocardial infarction, miR-185 levels show a biphasic pattern, initially decreasing and then increasing at discharge (Park et al. 2021). It has been demonstrated that MyD88 expression is dramatically enhanced in cardiomyocytes treated with hypoxia/reoxygenation (Ye et al. 2019). Another study has proven that MyD88 expression is markedly raised in I/R-injured animals (Zhang et al. 2017a). Furthermore, our study confirmed that LINC00461 bound to miR-185-3p and Myd88 was targeted by miR-185-3p. It has been shown that LINC00461 functions as a competing endogenous RNA of miRs (Chen et al. 2019; Deng et al. 2019). Studies have confirmed miR-induced targeting of MyD88 (Cao et al. 2019; Ma et al. 2019). While the relationship between LINC00461 and miR-185-3p as well as Myd88 and miR-185-3p has not been elucidated.

In addition, it was revealed in our study that depleted LINC00461 or restored miR-185-3p decreased LVEDP, LDH, CK-MB, cTnӀ, ROS and MDA levels, and enhanced LVEF, LVFS, ± dp/dt max, NO and SOD levels, as well as attenuated fibrosis and cardiomyocyte apoptosis in I/R mice. It has been suggested previously that LINC00461 down-regulation notably inhibits proliferation and promoted apoptosis of multiple myeloma cells (Deng et al. 2019). Moreover, a current publication has outlined that LINC00461 knockdown suppresses proliferation and migration of non-small cell lung cancer cells (Meng et al. 2020). Another study has verified that miR-185 up-regulation suppresses endoplasmic reticulum stress-caused apoptosis in the heart (Kim et al. 2016). It has been implicated that miR-185 could protect mice against myocardial infarction through promoting cardiac function recovery and attenuating cardiomyocyte apoptosis (Li et al. 2020). LDH is an critical enzyme that produces energy in hypoxia through anaerobic glycolysis (Leyva-Carrillo et al. 2019). The excessive production of ROS is usually associated with inflammation or cancer as well as may cause tissue damage (Bouche et al. 2019). Creatine kinase, which located in mitochondria and plasma of tissues including brain tissue, skeletal muscle, and cardiac muscle, is a key kinase correlated with ATP regeneration, intracellular energy transportation and muscle contraction and is widely utilized as a biomarker of myocardial injury (Zou et al. 2017). Indeed, LVEF and LVFS increases are the signals of improvement of myocardial I/R injury after treatment (Liu et al. 2016). A study revealed that in myocardial I/R insult, LVEF and + dp/dtmax decrease, LVEDP, serum CK, LDH, and cTnT levels increase, SOD activity reduces and MDA level raises in the myocardial tissue (Zhang et al. 2018a). In addition, it is displayed that LDH, ROS and MDA are decreased and SOD is increased by treatment in myocardial tissues (Pu et al. 2019). It has been documented that myocardial I/R results in a distinct increase in CK-MB, MDA and LDH activities, markedly a decrease in SOD and NO levels (Zhang et al. 2018b).

## Conclusion

In short, this investigation reveals that depleted LINC00461 alleviates myocardial I/R injury via suppressing miR-185-3p-targeted regulation of Myd88. LINC00461 may serve as a potential target for the treatment of myocardial I/R injury. However, a conclusion about the effects of LINC00461 cannot be made clearly due to limited exploration. It needs monitoring rigorously and reporting appropriately in the future clinical trials.

## Supplementary Information

**Additional file 1: Figure S1.** Up-regulating miR-185-3p or down-regulating LINC00461 attenuates heart dysfunction of I/R mice. A, Comparison of LVEF in each group of mice. B, Comparison of LVFS in each group of mice. C, Comparison of LVEDP in each group of mice. D, Comparison of + dp/dt max in each group of mice. E, Comparison of -dp/dt max in each group of mice. a *P* < 0.05 vs. the sham group. b *P* < 0.05 vs. the si-NC group. c *P* < 0.05 vs. the mimic-NC group. d *P* < 0.05 vs. the OE-LINC00461 + mimic NC group.

**Additional file 2: Figure S2** Bioinformatics prediction results. A, Binding sites between LINC00461 and miR-185-3p. B, Binding sites between MYD88 and miR-185-3p.

**Additional file 3: Table S1.** Primer sequences for qPCR.

## Data Availability

The original contributions presented in the study are included in the article/Supplementary Material, further inquiries can be directed to the corresponding author.

## Abbreviations

lncRNA: Long non-coding RNA
I/R: Ischemia–reperfusion
miR-185-3p: MicroRNA-185-3p
Myd88: Myeloid differentiation primary response gene 88
ECG: Electrocardiogram
NC: Negative control
OE: Overexpression
LVEDV: Left ventricular end diastolic volume
LVESV: Left ventricular end systolic volume
LVEF: Left ventricular ejection fractions
LVFS: Left ventricular fractional shortening
RT-qPCR: Reverse transcription quantitative polymerase chain reaction
GAPDH: Glyceraldehyde phosphate dehydrogenase
PBS: Phosphate buffered saline
PVDF: Polyvinylidene fluoride
WT: Wild type
MUT: Mutant type
UTR: Untranslated region
HE: Hematoxylin–eosin
TEM: Transmission electron microscope
TTC: Triphenyltetrazolium chloride
LAD: Left anterior descending coronary artery
TUNEL: Transferase-mediated dUTP-biotin nick end-labeling
OCT: Optimal cutting temperature
LDH: Lactate dehydrogenase
CK-MB: Creatine kinase-MB
cTn-I: Cardiac troponin I
NO: Nitric oxide
OD: Optical density
EDTA: Ethylene diamine tetraacetic acid
ROS: Reactive oxygen species
MDA: Malondialdeyde
ANOVA: Analysis of variance
