# Supplementary figures and images for: LncRNA LINC00461 exacerbates myocardial ischemia–reperfusion injury via microRNA-185-3p/Myd88
Source: Mol Med. 2022 Mar 10;28:33. doi: 10.1186/s10020-022-00452-1 (PMC8908691; doi:10.1186/s10020-022-00452-1)

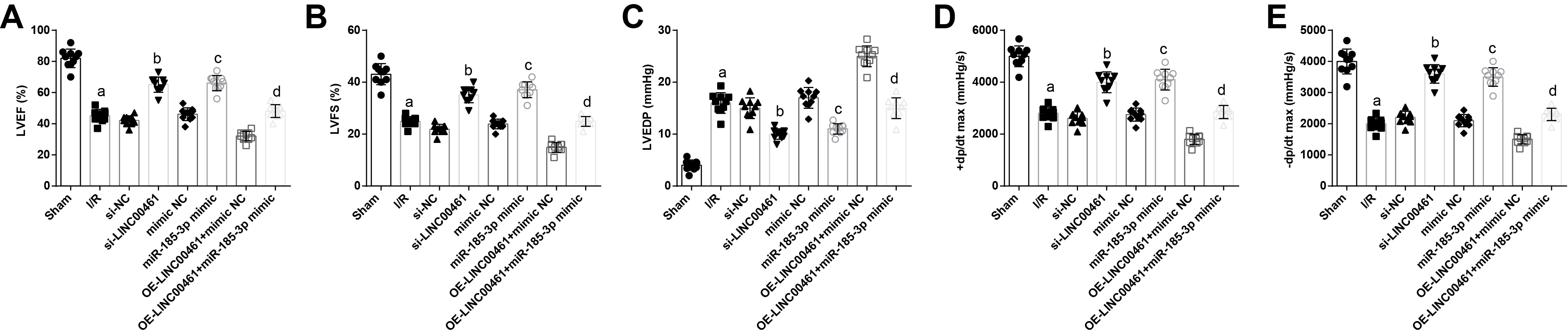

Supplement: Supplementary file 1 — Additional file 1: Figure S1. Up-regulating miR-185-3p or down-regulating LINC00461 attenuates heart dysfunction of I/R mice. A, Comparison of LVEF in each group of mice. B, Comparison of LVFS in each group of mice. C, Comparison of LVEDP in each group of mice. D, Comparison of + dp/dt max in each group of mice. E, Comparison of -dp/dt max in each group of mice. a P < 0.05 vs. the sham group. b P < 0.05 vs. the si-NC group. c P < 0.05 vs. the mimic-NC group. d P < 0.05 vs. the OE-LINC00461 + mimic NC group. [file 10020_2022_452_MOESM1_ESM.jpg]

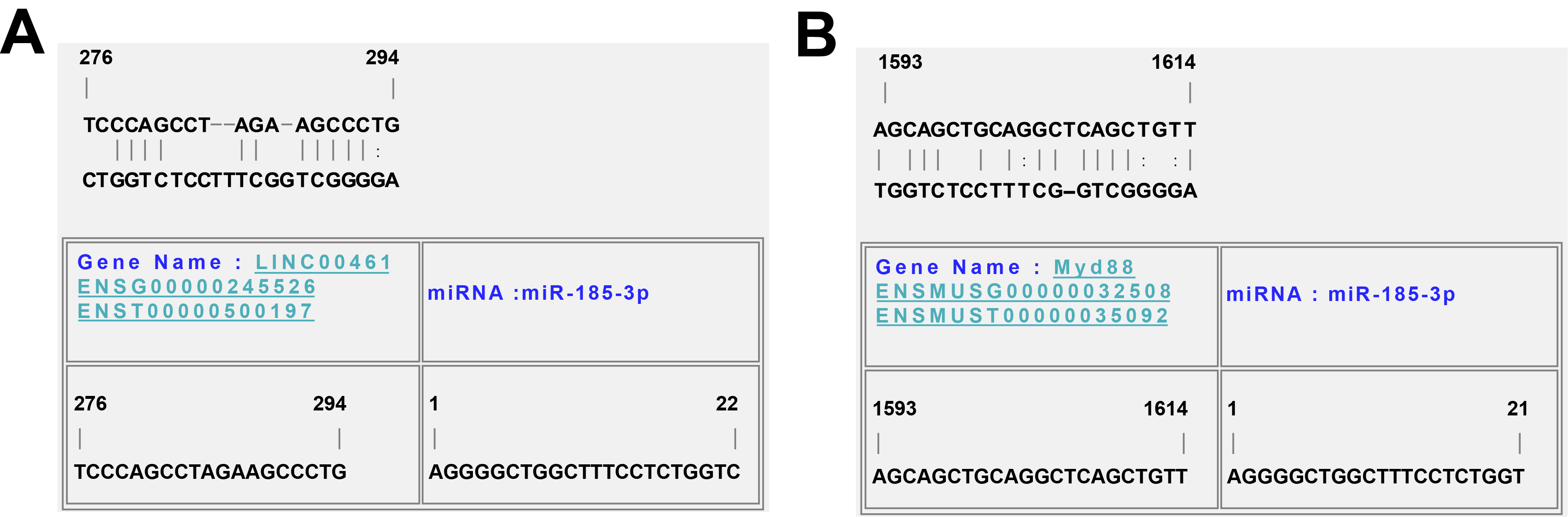

Supplement: Supplementary file 2 — Additional file 2: Figure S2 Bioinformatics prediction results. A, Binding sites between LINC00461 and miR-185-3p. B, Binding sites between MYD88 and miR-185-3p. [file 10020_2022_452_MOESM2_ESM.jpg]
