# Supplementary material for: LncRNA LINC00461 exacerbates myocardial ischemia–reperfusion injury via microRNA-185-3p/Myd88
Source: Mol Med. 2022 Mar 10;28:33. doi: 10.1186/s10020-022-00452-1 (PMC8908691; doi:10.1186/s10020-022-00452-1)
Supplement: Supplementary file 3 — Additional file 3: Table S1. Primer sequences for qPCR. [file 10020_2022_452_MOESM3_ESM.docx]

**Supplementary Table 1** Primer sequences for qPCR

| Gene | Sequence (5’→3’) | Temperature | Accession number |
| --- | --- | --- | --- |
| LINC00461 | F: TCTCTGTTCCAAGAGGGTTTCC | 59.6 | NR-152236.1 |
|  | R: GCTGTTTCCTGGATAGACCTGAT |  |  |
| miR-185-3p | F:AGGGGCTGGCTTTCCTCTGGT | 48.9 | NR_029571.1 |
| Myd88 | F: GCTACTGCCCCAACGATATC | 60.6 | NM_010851.3 |
|  | R: ACACAACTTAAG CCGATAGTCTG |  |  |
| U6 | F: ATTGGAACGATACAGAGAAGATT | 55.6 | XR_004933974.1 |
|  | R: GGAACGCTTCACGAATTTG |  |  |
| GAPDH | F: ATGCTGCCCTTACCCCGG | 62.6 | XM_036165840.1 |
|  | R: TTACTCCTTGGAGGCCATGTAGG |  |  |

Note: F, forward; R, reverse; LINC00461, long non-coding RNA LINC00461; miR-185-3p, microRNA-185-3p; Myd88, myeloid differentiation primary response gene 88; GAPDH, glyceraldehyde phosphate dehydrogenase.
